# Juxtaglomerular cell tumor: report of a case with unusual presentation

**DOI:** 10.4322/acr.2021.406

**Published:** 2022-10-21

**Authors:** Priscilla Quach, Ameer Hamza

**Affiliations:** 1 University of Kansas Medical Center, Department of Pathology & Laboratory Medicine, Kansas City, Kansas, USA

**Keywords:** Glomus tumor, Renin, Aldosterone, Juxtaglomerular apparatus, Hypertension

## Abstract

Juxtaglomerular cell tumor is a benign, renin-secreting neoplasm. The tumor arises from the juxtaglomerular apparatus cells of the kidney. Because the tumor is hormonally active, patients usually suffer from hypokalemia, hyperaldosteronism, and hypertension. Herein, we describe a case of a 19-year-old Asian female with a somewhat unusual presentation.

A 19-year-old Asian female presented with upper extremity weakness, numbness, and tingling. On physical examination, the only notable finding was hypertension. Extensive workup revealed elevated aldosterone level and plasma renin activity. CT scan of the abdomen revealed a 2.2 cm mass in the lower pole of the left kidney. The mass was resected by partial nephrectomy. On microscopic evaluation, the tumor had glomoid appearance with sheets of uniform, round to polygonal cells with clear to eosinophilic cytoplasm. Immunohistochemical stains showed the tumor cells to be positive for CD117, CD34 and CD10 and negative for ER, PR, CK7, PAX-8, pan-cytokeratin, EMA, S100, Melan-A, HMB45, SMA and CAIX. Diagnosis of Juxtaglomerular cell tumor was rendered. This case highlights the importance of a regular physical exam and a high index of suspicion in patients presenting with unusual complaints.

## INTRODUCTION

Juxtaglomerular cell tumor (JGCT) is a benign, renin-secreting neoplasm.^1^ The tumor arises from the juxtaglomerular apparatus cells of the kidney.^1^ Because the tumor is hormonally active, patients usually suffer from hypokalemia, hyperaldosteronism, and hypertension.^1^^-^ 4 The typical patient population is adolescents and young adults, with a slight predominance in females.^1^^-^ 4 The most common clinical presentation is that of a young adult with severe hypertension refractory to medical therapy. Elevated levels of aldosterone and renin coupled with a renal mass on imaging are the mainstay of diagnosis, which is eventually confirmed histologically following resection of the mass.^2^^-^ 4 Herein, we describe a case of a 19-year-old Asian female with a somewhat atypical presentation. This case highlights the importance of a regular physical exam and having a high index of suspicion in patients presenting with unusual complaints.

## CASE REPORT

**Figure 1:**
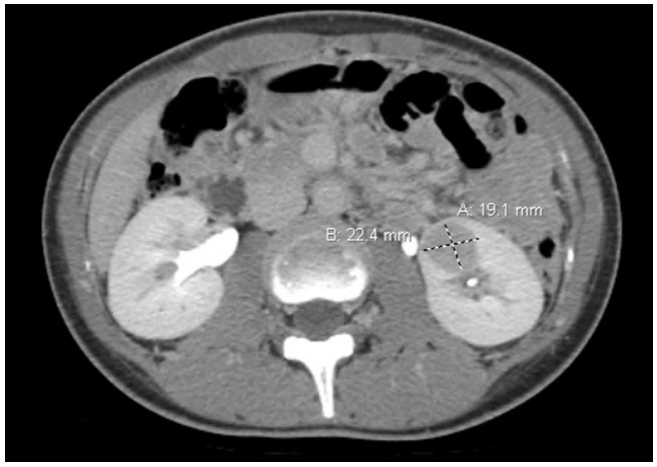
Abdominal CT scan, axial plane, demonstrating the left renal mass.

A 19-year-old Asian female presented with upper extremity weakness, numbness, and tingling. On physical examination, the only notable finding was hypertension (blood pressure 150/90), which the patient was not aware of. Other physical exam, including neurologic exam and indirect ophthalmoscopic exam, showed no abnormalities. Electrocardiogram revealed normal sinus rhythm and no abnormalities. Because hypertension is unusual in young adults, a systematic investigation was initiated. The patient was mildly anemic with hemoglobin of 11.7 gm/dL (Normal range: 12-15 gm/dL), all other complete blood count values were within normal limits. Comprehensive metabolic panel was also within normal limits, although serum potassium was at the lower end of the normal range at 3.5 mmol/L (Normal range: 3.5-5.1 mmol/L). Plasma and urinary metanephrines and catecholamines were also within the normal range. Additional hormonal workup revealed an aldosterone level of 71 ng/dL (Normal range: 3-24 ng/dL) and plasma renin activity of 6.25 ng/mL/hr (Normal range: 0.6 to 4.3 ng/mL/hr. Thyroid hormone levels were within normal limits. A CT scan of the abdomen revealed a 2.2 cm mass in the lower pole of the left kidney (Figure 1). There was no retroperitoneal adenopathy or evidence of metastatic disease. Clinically, the mass was suspected to be hormonally active and the underlying cause of patient's hypertension. The mass was surgically resected by partial nephrectomy.

The surgical pathology specimen consisted of a left partial nephrectomy (4.0 x 3.2 x 2.2 cm), including a scant amount of perinephric adipose tissue. There was a 2.4 x 1.8 x 1.7 cm well-circumscribed, encapsulated tumor in the kidney. The tumor was located less than 0.1 cm from the closest parenchymal resection margin and was confined to the kidney.

**Figure 2:**
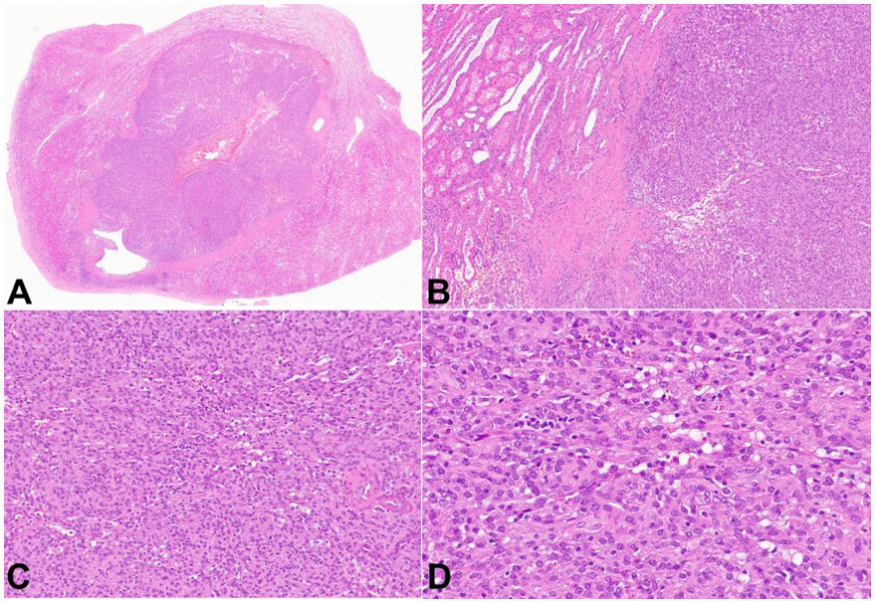
Photomicrographs of the tumor. **A** – Wholemount showing cross-section of the tumor (H&E 4x); **B** – Tumor in relation to renal parenchyma (H&E 40x); **C** – Medium power photomicrograph showing cytologic details (H&E 100x); **D** – Tumor composed of uniform, round to polygonal cells with clear to eosinophilic cytoplasm (H&E 200x).

On microscopic evaluation, the tumor had glomoid appearance with sheets of uniform, round to polygonal cells with clear to eosinophilic cytoplasm and a dispersed lymphoplasmacytic infiltrate. The stroma was scant, mitoses were rare, and necrosis was not identified (Figure 2).

**Figure 3:**
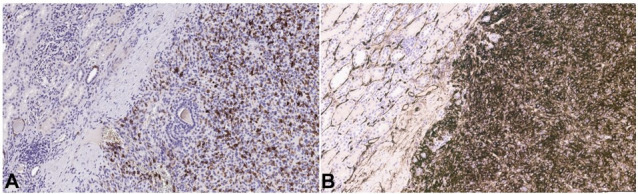
Photomicrographs of the tumor. **A** – Tumor cells are positive for CD117 (100x) and; **B** – CD34 (100x).

Immunohistochemical stains showed the tumor cells to be positive for CD117, CD34 and CD10 (Figure 3). They were negative for ER, PR, CK7, PAX-8, pan-cytokeratin, EMA, S100, Melan-A, HMB45, SMA and CAIX.

Based on the morphology and immunohistochemical profile, diagnosis of Juxtaglomerular cell tumor was rendered.

The patient was alive and well and without signs of recurrence 14 months after surgery.

## DISCUSSION

Juxtaglomerular cell tumor is a rare, benign, renin-secreting renal neoplasm. It results from dysfunction of the juxtaglomerular cells of the kidney, leading to over-expression of renin which in turn increases aldosterone levels. This hyperaldosteronism causes the typical symptoms seen with this entity, including hypertension and hypokalemia. Other reported symptoms include headache, vomiting, dizziness, nocturia, and polyuria.^2^^-^ 4 Data from published cases and reviews suggest that the tumor frequently affects adolescents and young adults with a mean age 27 years at diagnosis and is twice as common in women as in men. Although the tumor mainly affects young adults, cases have been reported in children and the elderly with an age range of 6-69 years.^1^^,^ 4 ^-^ 6 The prototypical presentation of a young female with unexplained hypertension was also seen in our case. However, the presenting symptoms of upper extremity weakness, numbness, and tingling were somewhat unusual. Although these symptoms can be explained by hypokalemia, most previous studies have not reported these symptoms.^2^^-^ 4

Workup of hypertension in a young patient always begins with a comprehensive history and physical exam. The finding of persistent hypertension requires blood work and imaging studies. The most common cause of secondary hypertension in young adults, especially females, is renal artery stenosis caused by fibromuscular dysplasia.^7^ This is best investigated using ultrasonography or CT imaging. In regard to diagnosing JGCT, both of these modalities may also be used. Of the two, CT is the more sensitive choice.^4^^,^ 7 While the findings are non-specific (small solitary tumor confined to the kidney), it’s still the preferred modality for detecting a JGCT. Enhanced CT studies are preferred as iso-dense tumors may be missed on unenhanced imaging.^8^ MRI imaging has also been utilized in JGCT. The reported features include iso-intensity or mild hyperintensity on T2-weighted imaging, heterogeneous hyperintensity on diffusion-weighted imaging, and a degree of enhancement <200% in the corticomedullary phase.^9^ The tumor size typically ranges from 2-5 cm as was seen in our case with the tumor measuring 2.4 cm in greatest dimension, but larger sizes have been reported. Imaging results demonstrating a renal mass in younger individuals usually prompts testing of aldosterone and renin levels if these are not tested in the early stages of the workup.^7^

Grossly the tumors are well-encapsulated, unilateral, and solitary. Histologically, the tumor cells have a glomoid appearance being composed of sheets of round to polygonal cells with slightly eosinophilic cytoplasm.^1^^,^ 4 Rarely, the tumors may have a papillary growth pattern 10. Mitoses are infrequent, and focal necrosis can also be identified in some cases. Most tumors also demonstrate a prominent vasculature consisting of thin- and thick-walled capillaries along with branching blood vessels and sinusoids reminiscent of hemangiopericytoma. In fact, juxtaglomerular cell tumors were originally classified as hemangiopericytomas because of their prominent vascular network^11^^-^ 13. Mast cells in varying numbers and a lymphoplasmacytic infiltrate may also be seen.^2^^,^ 3 Our case showed the glomoid appearance, lymphoplasmacytic infiltrate, and rare mitoses. Immunohistochemically these tumors are frequently positive for CD34 and SMA.^2^^,^ 4 Additionally, they are immunoreactive to renin and CD117.^3^ It is noteworthy that, unlike many other renal tumors, JGCTs are negative for PAX-8.

From a pathologic standpoint, the most important differential considerations include glomus tumor, solitary fibrous tumor, and epithelioid angiomyolipoma. Distinction from glomus tumors is extremely challenging because of the considerable morphologic overlap between the two entities; however, immunohistochemistry is very helpful. JGCTs are frequently positive for renin, CD34, and CD117, whereas glomus tumors are negative or only focally positive for CD34 and are negative for renin and CD117.^14^^-^ 16

In solitary fibrous tumor, the cells are usually more spindled. Additionally, the tumor cells are immunoreactive to CD99, Bcl-2, and STAT6, and negative for renin.^14^

Although JGCT can show variable positivity for SMA they are negative for melanocytic markers including HMB45 and Melan-A, unlike epithelioid angiomyolipoma.^17^^,^ 18

Like other renal tumors, surgery is the primary treatment modality and is usually done with curative intent. Depending on several factors, including tumor size and location, either partial or radical nephrectomy can be performed. Patients usually report improvement in the symptoms, including hypertension, after the surgery; however, a small subset may continue to have hypertension following resection.^4^

Most JGCTs follow an indolent course with an excellent prognosis; however, a rare case with metastasis has been reported in the literature.^19^
